# Shaping and Controlled Fragmentation of Liquid Metal Droplets through Cavitation

**DOI:** 10.1038/s41598-017-19140-w

**Published:** 2018-01-12

**Authors:** M. S. Krivokorytov, Q. Zeng, B. V. Lakatosh, A. Yu. Vinokhodov, Yu. V. Sidelnikov, V. O. Kompanets, V. M. Krivtsun, K. N. Koshelev, C. D. Ohl, V. V. Medvedev

**Affiliations:** 1 Moscow Institute of Physics and Technology (State University), Institutskiy pereulok str. 9, Dolgoprudny, Moscow region, 141701 Russia; 2 Institute for Spectroscopy RAS, Fizicheskaya str. 5, Troitsk, Moscow 108840 Russia; 3 Division of Physics and Applied Physics, School of Physical and Mathematical Sciences, Nanyang Technological University, 21 Nanyang Link, Singapore, 637371 Singapore; 4 RnD-ISAN/EUV Labs, Sirenevy Bulevard Str. 1, Troitsk, Moscow 108840 Russia; 5 Department for Soft Matter, Fakultät für Naturwissenschaften, Otto-von-Guericke-Universität Magdeburg, Universitätsplatz 2, 39106 Magdeburg, Germany

## Abstract

Targeting micrometer sized metal droplets with near-infrared sub-picosecond laser pulses generates intense stress-confined acoustic waves within the droplet. Spherical focusing amplifies their pressures. The rarefaction wave nucleates cavitation at the center of the droplet, which explosively expands with a repeatable fragmentation scenario resulting into high-speed jetting. We predict the number of jets as a function of the laser energy by coupling the cavitation bubble dynamics with Rayleigh-Taylor instabilities. This provides a path to control cavitation and droplet shaping of liquid metals in particular for their use as targets in extreme-UV light sources.

## Introduction

Cavitation and bubble dynamics in liquid metals are a challenge to experimental studies as conventional tools such as high-speed photography or acoustic detection with hydrophones are not possible. In order to directly observe cavitation bubbles in bulk liquid metals complex experiments employing pulsed X-ray imaging using e.g. synchrotron radiation sources are required^1^. The properties of metals such as their high densities at low vapour pressure combined with a large surface tension coefficient makes them particularly interesting for cavitation bubble dynamics studies. For example it was revealed^2,3^ that sonoluminescence can be induced in liquid metals by creating cavitation through acoustic excitation. The dynamics of cavitation bubbles particularly in mercury has received attention after large scale cavitation erosion of structural elements were observed in spallation neutron sources^4,5^. While cavitation plays an important role in various technological processes of modern metallurgy^6–8^ it also allows to probe for tensile strength thresholds^9–12^.

In the present report we reveal for the first time the mechanism by which a laser pulse transforms an initial spherical liquid metal droplet into an expanding droplet decorated with multiple radial jets. The key finding is that the laser pulse nucleates by acoustic means a cavitation bubble at the center of the droplet. During the volume expansion of this cavitation bubble the droplet surface becomes unstable and develops thin jets. We support this observation with a model based on a spherical expanding bubble able to predict this instability and the number of jets created.

## Results and Discussion

**Figure 1:**
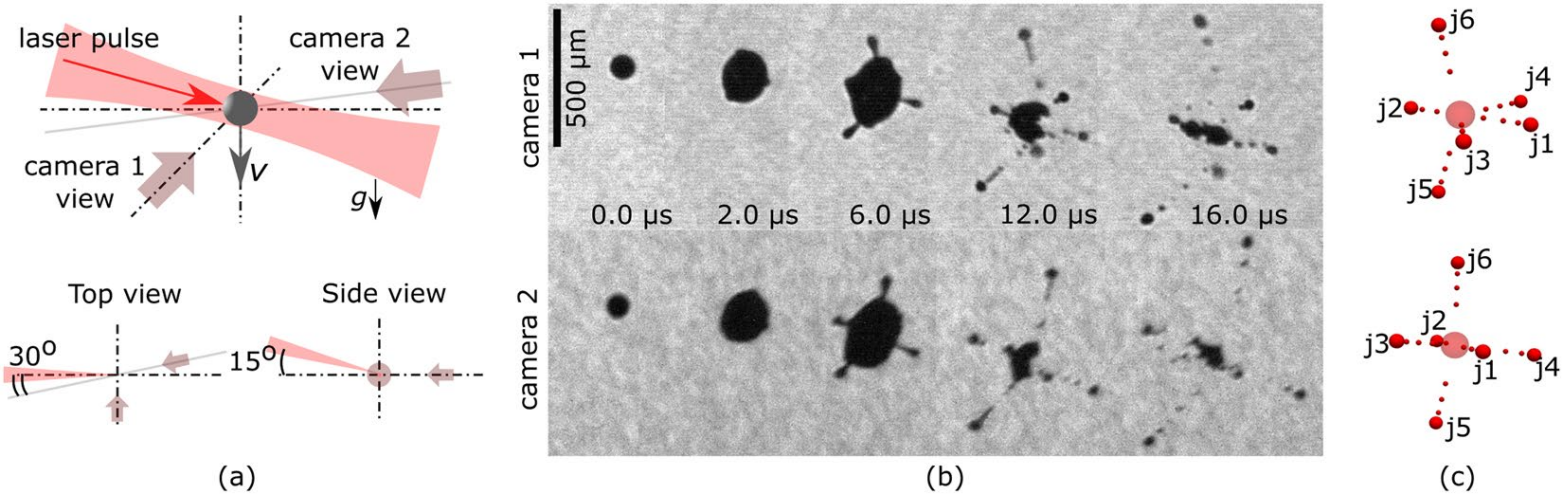
(**a**) The geometry of the experiment. (**b**) The shadowgraphs (top - camera 1 (side-view), bottom - camera 2 (front-view)) of deformed droplets taken at different time delays *t* after the laser pulse impact; the laser pulse energy equals 0.83 mJ; each frame corresponds to a separate experimental realization. (**c**) Analysis of the fragments’ positions.

Figure 1 depicts a typical experimental result following the exposure of the metal droplet with a short laser pulse (see Methods section) with an energy of 0.83 mJ (5.8 ⋅ 10^12^ W/cm^2^). Snapshots of the initial droplet (first frame) and its shape transformation after the laser impact are shown in Fig. 1b (view of camera 1 is shown in the upper row, camera 2 below). The drop expands for the first 2 *μ*s following the laser pulse. Assuming that the density of the liquid remains unchanged, conservation of mass dictates that the expansion is due to void creation within the droplet, e.g. the expansion of the droplet into a thin shelled object. Interestingly, the initial smooth surface of the expanding droplet displays humps already visible at *t* = 2 *μ*s. The droplet reaches its maximum size around *t* = 6 *μ*s; here the humps have transformed into slender jets which detach from the core droplet during the retraction of the shell visible from *t* = 12 *μ*s in Fig. 1b. The central shell collapses back into a small droplet while the jets move radially away and fragment further due to Rayleigh-Plateau instabilities^13,14^. The estimated size of the largest fragments from jets observed on the shadowgraphs is 20 *μ*m. The measured velocities of these fragments are of the order of 10 m/s.

We want to emphasize that the described evolution of the droplet shape upon the laser impact as well as the numbers of jets and their directions is very stable from pulse-to-pulse. By analyzing the perpendicular camera views at the same instant of time it is possible to obtain the position of the jets. Figure 1c depicts such an analysis for *t* = 12 *μ*s for the upper and lower row, respectively. In total we find 6 jets which are numbered from *j*_1_ to *j*_6_. Two jets *j*_1_, *j*_2_ move in opposite directions along the laser beam axis. The remaining four jets can be separated in two jets each (*j*_3_, *j*_4_) and (*j*_5_, *j*_6_) propagating in perpendicular planes, which cross each other on the laser beam axis.

What causes the nucleation of cavitation leading to the droplet expansion? Pulsed laser loading is a well-known method of generation of stress waves in condensed matter^15–20^. The directed laser energy deposition first produces a compression pulse or a shockwave. Diffraction waves at the edges of the illuminated region trail the compressive wave as a rarefaction wave and subjects the droplet to tensile stress. Once the tensile stress is above the yield strength of the liquid it ruptures and a vapor cavity is formed. These phenomena were extensively studied for planar target geometries for various laser parameters and target materials. Typically liquids have yield strength of thousand bar of negative pressures. The question arises what causes such large amplitudes. In the present experiments laser intensities of 10^12^–10^13^ W/cm^2^ in the focal plane are achieved. These are well above the ablation threshold for tin^21^. Due to the ablation process the laser pulse launches a pressure pulse from the irradiated side of the droplet. According to the numerical simulations by Eidmann *et al*.^22^ pressure in the ablation front can reach magnitudes of Mbar-scale at our laser intensities. Note that the pressure is applied within a time scale, which is four orders of magnitude faster than the sonic time for the droplet, i.e. *t*_*s*_ ≈ *d*/*c*_*s*_ ≈ 25 ns. Such ultrashort and ultrastrong pressure kick inevitably ignites a shockwave at the laser-irradiated surface of the droplet target which then propagates through the target’s body. The significant factor that distinguishes our experiments from the classical studies with planar targets is the shape of the shockwave front. In this study, the target (droplet) geometry implies formation of a hemispherical shockwave. Furthermore, the shockwave converges, i.e. focuses, during its propagation through the droplet. There are two competing processes that determine the pressure magnitude at the front of the shockwave. The first process is the energy dissipation which leads to the decrease of pressure. The second process is the shockwave focusing, which acts in the opposite direction. By analogy with acoustic waves, the pressure enhancement factor due to the focusing can be estimated as *kRα*^23^, where *k* is the acoustic wave number, *R* is the radius of the focusing geometry, and $\alpha ({f}_{N})=1-\sqrt{1-\mathrm{1/(4}{f}_{N}^{2})}$ a geometric factor depending on the acoustic *f*-number; *α* ≈ 0.1 for the present geometry. With an estimate of the acoustic wavelength of the *c*_*s*_*τ* ≈ 2 nm we obtain an acoustic gain of the pressure within the droplet of *G* ≈ 1.5 ⋅ 10^4^. Such a high gain suggests that the focusing can compensate effect of the energy dissipation and can deliver the ultra-strong shockwave to the center of the droplet. The same considerations are applicable for the rarefaction wave that follows the shockwave. The rarefaction wave focused to the center of the droplet. There the magnitude of the negative pressure (tensile stress) becomes enhanced. When the magnitude of the negative pressure reaches the value of the tensile strength of the liquid a cavitation bubble in the metal droplet is nucleated. Note that the rupture of liquid may also occur from the reflection of the pressure wave at the droplets free interface (its back)^24,25^. However, in this study we operate the laser in an energy regime to avoid the latter phenomenon.

**Figure 2:**
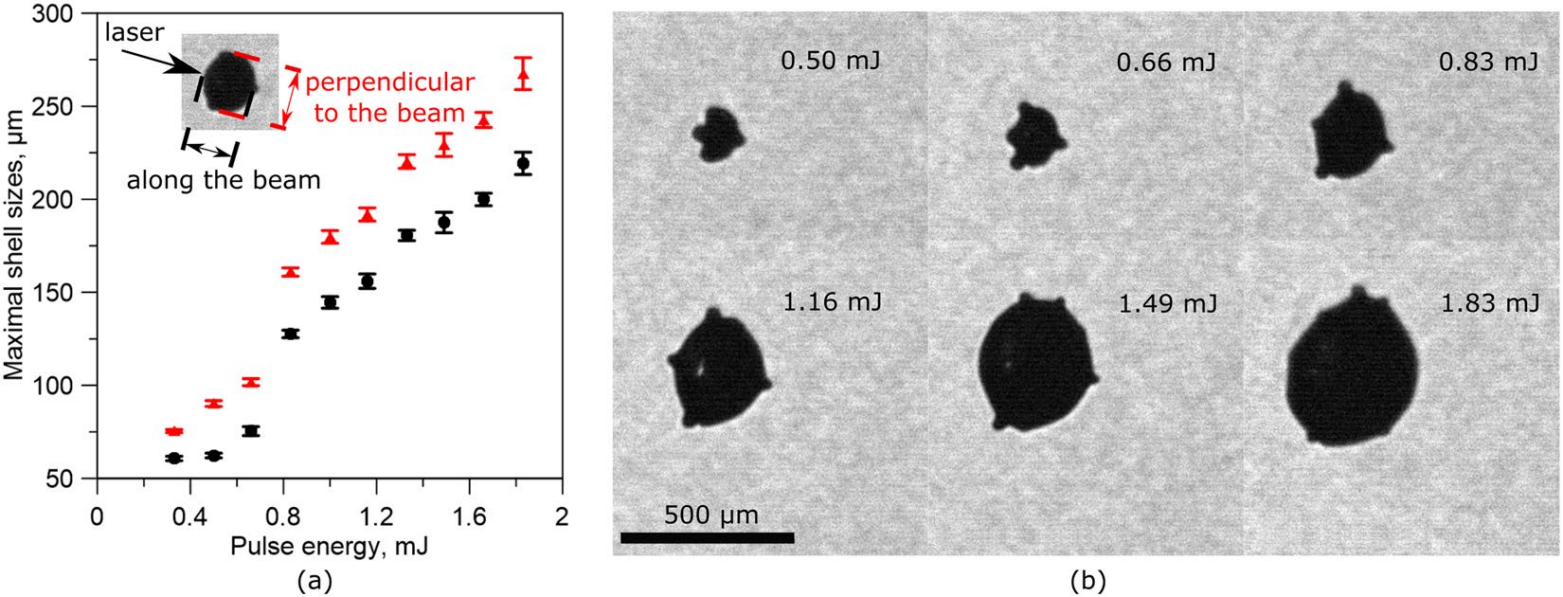
Experimental results. (**a**) Maximum expansion of the droplet in the direction (black symbols) and vertical (red symbols) to the laser beam. (**b**) Shadowgraphs of the droplet at maximum expansion.

**Figure 3:**
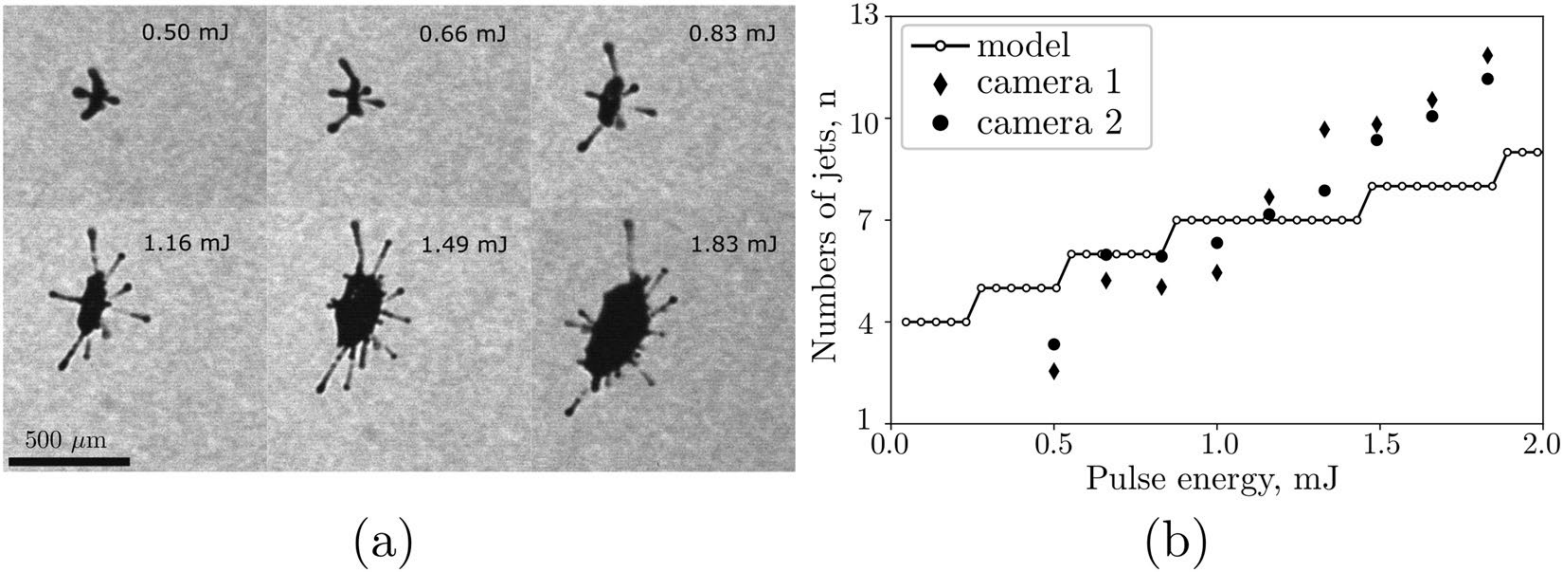
Experimental results. (**a**) Shadowgraphs of the droplet taken at 8 *μ*s after laser pulse. (**b**) Mean number of jets as a function of pulse energy for camera 1 view (diamond symbols) and camera 2 view (round symbols) both with the numerical result on the peak number of the most unstable surface wave.

Figure 2 reveals the importance of the laser energy controlling the size of the expanded droplet. For the smallest laser pulse energy used − 0.33 mJ (*I* = 2.3 ⋅ 10^12^ W/cm^2^) - we see only a mild expansion yet already here the surface is decorated with some humps. The diameter increases approximately linearly with the laser energy, see Fig. 2a. Interestingly, not only the volume of the droplet but also the number of humps increases, Fig. 2b; and so does the number of jets. The number of jets is counted in both camera views for 100 experiments, averaged and plotted in Fig. 3b as a function of the laser energy. Typical snapshots showing these jets are depicted in Fig. 3a. The pictures reveal that the directions of the jets are a function of the laser energy: for laser pulse energies below 1.33 mJ we find jets always along the six main jetting directions *j*1–*j*6. Above 1 mJ more jets form while their direction remains reproducible from shot-to-shot. Interestingly, the jets along the axis of the laser *j*1 and *j*2 become less pronounced while the jets in the perpendicular directions (*j*3–*j*4 and *j*5–*j*6) remain for all studied energies. From 1.49 mJ additional jets appear which vary from shot-to-shot. Thus for laser energy of 1.49 mJ and above the trajectories of the fragments are unpredictable.

Detailed understanding and quantitative analysis of the presented experimental studies requires a multiphysics model, which describes the processes of laser ablation, formation and propagation of the shockwave with account for its energy dissipation, thermodynamics of the metastable liquid and nucleation of cavitation bubbles, and the fluid dynamics of free-surface flow with fragmentation and heat transfer. Fortunately, to understand the mechanism destabilization of the interface already a greatly simplified model can be applied. Because the experiments revealed that the humps are formed during the early time of the bubble expansion, i.e. during times of high acceleration, we investigate next the stability of the outer droplet surface to this radial acceleration.

The model starts with a bubble of initial radius *R*_*b*0_ and uniform high pressure *P*_0_ being nucleated by the convergent rarefaction wave. It is located at the center of the droplet with an initial radius of *R*_0_. The droplet is placed in vacuum, thus the retraction force leading to the shrinkage of the droplet is surface tension only. The bubble *R*_*b*_(*t*) and droplet *R*(*t*) radii are related through conservation of volume: $R{(t)}^{3}-{R}_{b}{(t)}^{3}={R}_{0}^{3}-{R}_{b0}^{3}$. The spherical bubble dynamics is modeled with a modified Rayleigh-Plesset equation similar to^26^, two surface tension terms have been added, which are causing the bubble collapse:

1 $$\frac{{P}_{0}{R}_{b0}^{3\gamma }}{{R}_{b}^{3\gamma }\rho }=\frac{3}{2}{\dot{R}}_{b}^{2}+{R}_{b}{\ddot{R}}_{b}+\frac{4\mu }{{R}_{b}\rho }{\dot{R}}_{b}+\frac{2\sigma }{\rho {R}_{b}}+\frac{2\sigma }{\rho R}-2{\dot{R}}_{b}^{2}\lambda -{R}_{b}{\ddot{R}}_{b}\lambda +\frac{1}{2}{\dot{R}}_{b}^{2}{\lambda }^{4},$$

with *λ* = *R*_*b*_(*t*)/*R*(*t*). Here *ρ* = 7.3 g⋅cm^−3^ and *μ* = 1.75 × 10^−3^ kg⋅m^−1^s^−1^ are the density and dynamic viscosity of the liquid tin alloy, *σ* = 0.534 N/m is the coefficient of surface tension. The model assumes that the droplet remains a sphere, this allows to model the initial part of the bubble expansion, yet once the jets are formed the model may only provide a qualitative description of the gross dynamics of the droplet. Yet, the humps are formed during the early expansion phase which is unstable to Rayleigh-Taylor instabilities, see ref.^27^.

To compare the simulation with the experiment we model the number of jets as a function of laser energy by identifying the humps with the fastest growing modes of the spherical surface perturbations. These surface waves can be described with spherical harmonics *Y*_*n*_ of mode *n*, i.e. $A(t)=\sum _{n\mathrm{=2}}^{\infty }{a}_{n}(t){Y}_{n}$. Here *n* is the mode number and also the peak number of each surface wave; *a*_*n*_ is the amplitude of the spherical harmonic of mode *n*, starting from small initial value $|{a}_{n}(t=\mathrm{0)}|\ll {R}_{0}$, its evolution equation to the first order for incompressible and viscous spherical flows can be obtained from^28^:

2 $${\ddot{a}}_{n}+{B}_{n}(t){\dot{a}}_{n}-{A}_{n}(t){a}_{n}=0$$

with

3 $${A}_{n}(t)=-(n+\mathrm{2)}\frac{\ddot{R}}{R}-(n-\mathrm{1)}n(n+\mathrm{2)}\frac{\sigma }{\rho {R}^{3}}-(n-\mathrm{1)(}n+\mathrm{2)}\frac{2\mu \dot{R}}{\rho {R}^{3}}$$

and

4 $${B}_{n}(t)=\frac{3\dot{R}}{R}+\frac{\mathrm{2(}n-\mathrm{1)(2}n+\mathrm{1)}\mu }{\rho {R}^{2}}\mathrm{.}$$

**Figure 4:**
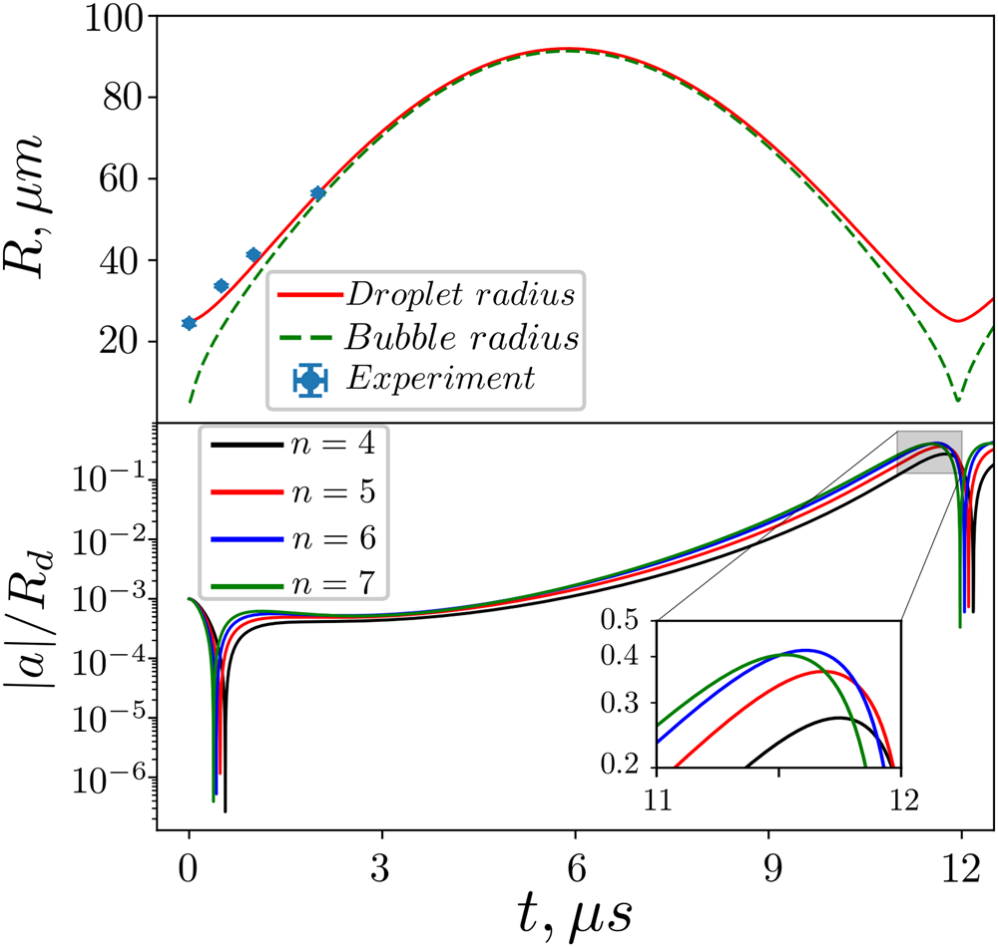
Numerical results. Top: the comparison of experimental and numerical results, the dots represent the droplet radius in experiment under pulse energy of 0.83 mJ, the solid line and the dash line are the numerical result of the droplet and bubble radius evolution with the initial condition of *R*_0_ = 25 *μm*, *R*_*b*0_ = 5 *μm*, *P*_0_ = 900 *bar*. Bottom: the normalized amplitude of the perturbations of different modes.

Firstly we calculated the time dependent droplet radius and bubble radius with Eq. (1). The initial conditions are *R*_0_ = 25 *μm*, *R*_*b*0_ = 5 *μm*, *P*_0_ = 900 *bar*. The numerical result is displayed in the top of Fig. 4 together with the experimental droplet radius for a pulse energy of 0.83 *mJ*. Good agreement is achieved, even the collapse time in the experiment, ~12 *μ*s, is reproduced indicating the importance of surface tension driving the droplet shrinkage. Based on this comparison we can estimate the converted fraction of the laser energy deposited on the droplet to the initial bubble energy *η* = *P*_0_*V*_*b*0_/*E* ≈ 10^−4^. The amplitude of each surface perturbations *a*_*n*_(*t*) can be obtained by combining Eqs (2) and (1) to obtain the most unstable surface wave and its number of positive peaks. This is plotted in Fig. 3b as a function of laser energy. We find excellent agreement with the experimental observation considering the simplicity of the model and complex acoustics and fluid mechanics leading to jetting. At high pulse energy the peak number is slightly underestimated, which may be explained with the excitation of more than one surface wave or the limits of the linear analysis on which Rayleigh-Taylor instabilities are based on.

### Summary

1) Dynamics of the cavitation bubbles inside liquid metal droplets was experimentaly studied. Cavitation was incepted using the laser-generated shockwaves. This finding is of interest for studies of the interaction of shockwaves with condensed matter. The latter usually consider shocks with plane wavefront propagating in planar samples, e.g. plates. Under such conditions rupture of matter occurs due the interaction of the shockwave with a free surface (boundary) of the sample, i.e. due to the spallation effect. Here we describe the experimental conditions when spallation is suppressed and rupture occurs within the bulk of the liquid. 2) Expansion and collapse of the droplet with bubble inside was observed. During the expansion the outer droplet surface becomes Rayleigh-Taylor unstable which leads to the ejection of jets. These instabilities were analyzed using the adopted Rayleigh-Plesset model. The model simulations were found to be in good agreement with the experimental observations. 3) The reported results provide useful tests for numerical models of strongly compressible multiphase flows accompanied by cavitation and fragmentation^29–36^ and for equation of states of liquid metals at extreme conditions^37–40^. Besides the fundamental interest, the findings may help for the optimization of industrial sources for extreme ultraviolet radiation. The hollow droplets as they are created by the expanding cavitation bubble may be an alternate to currently used target morphologies for extreme ultraviolet radiation^41–44^, in particular as the fragmentation scenario is highly repeatable and predictable.

## Methods

Liquid metal droplets of Sn-In eutectic (48–52% mass stoichiometry) alloy were generated with an in-house-made droplet generator utilizing stimulated jet breakup. Technical specification of the droplet generator can be found in our previous publication^45^. The temperature of the alloy in the system was maintained at 140 °C, which is 20 °C higher than the melting temperature of the Sn-In eutectic. All droplets studied in this paper had a diameter of 49.0 ± 1.6 *μ*m. Their vertical velocity was 9 m/s. The nozzle of the droplet generator was mounted within a vacuum chamber at a residual pressure of <10^−4^ mbar. The droplets remained liquid during the flight inside the vacuum chamber due to weak radiative cooling at the working temperatures. To initiate the complex flow inside the droplets, they were irradiated by pulses from a mode-locked Ti:sapphire laser (Spectra Physics, *λ* = 780–820 nm). The pulse duration was 0.8 ps (FWHM). The laser beam diameter at the focal plane was 150 *μ*m (FWHM) The laser pulse energy was varied between 0.33 mJ and 1.83 mJ. The laser was directed under a small angle of 15° to the horizontal, see Fig. 1a. The fragmentation scenarios are highly repeatable which allows stroboscopic shadow photography for visualization of the dynamics. 30-ns-long pulses from diode laser operating at the wavelength of 850 nm were used for the instantaneous back-lighting for the shadow photography. The shadow images were recorded using two cameras equipped with long distance microscopes and CCD arrays. The cameras under nearly perpendicular view observed the resulting droplet dynamics.
